# Coronavirus disease 2019 (COVID-19) polymerase chain reaction (PCR) screening of asymptomatic healthcare workers in a low-prevalence setting: A single-center UK cohort study

**DOI:** 10.1017/ash.2022.44

**Published:** 2022-05-23

**Authors:** Tariq Azamgarhi, Laura Maynard-Smith, Hannah Bysouth, Simon Warren

**Affiliations:** 1 Pharmacy Department, Royal National Orthopaedic Hospital NHS Trust, Brockley Hill, Stanmore, United Kingdom; 2 The Royal Free Hospital NHS Foundation Trust, London, United Kingdom; 3 Infection Prevention and Control Department, Royal National Orthopaedic Hospital NHS Trust, Brockley Hill, Stanmore, United Kingdom

**Keywords:** COVID-19, Healthcare workers, Screening

## Abstract

In this cohort study of UK healthcare workers, we evaluated the use of fortnightly polymerase chain reaction (PCR) screening to facilitate the safe resumption of elective surgery in a low-prevalence setting. We found that adherence to serial testing was poor, and the resource required to identify 1 asymptomatic case was substantial.

The coronavirus disease 2019 (COVID-19) pandemic has caused major disruption to elective surgery. To facilitate its safe resumption, healthcare worker (HCW) screening has become commonplace; however, the optimal strategy has not been defined.

We describe the use of fortnightly polymerase chain reaction (PCR) screening for severe acute respiratory coronavirus virus 2 (SARS-CoV-2), adherence to serial testing, and the resources required during a period of low community prevalence.

## Methods

This study was conducted at a tertiary-care orthopedic hospital in the London region over 8 weeks of low community prevalence of between 3.0 per 100,000 population on June 3 and 6.7 per 100,000 on July 27, 2020.^1^

Standard measures to prevent HCW transmission included mandatory use of surgical face masks and social distancing in accordance with national guidance. Workplace risk assessments were performed, and measures including reconfiguration of workspaces and changes to working patterns were implemented. Onsite HCWs were required to have a daily temperature check and to complete a 3-point symptom-based screening questionnaire. HCWs with symptoms or a fever of 37.8°C were instructed to self-isolate. They were referred to occupational health, and they underwent a combined nose and throat swab in accordance with Public Health England (PHE) guidance.^2^

All onsite HCWs were asked to attend fortnightly appointments for asymptomatic screening. Testing frequency was chosen based on the upper limit of the incubation period of 2–14 days. Adherence to serial screening was calculated based on the minimum frequency that HCWs would be required to attend fortnightly testing over the 8-week study period. HCWs with no test or who attended a single test were categorized as nonadherent; those who attended 2 or 3 tests were categorized as partially adherent; and those who attended 4 or more tests were categorized as fully adherent. Invalid tests for any reason were excluded from the analysis. All swabs were tested at a single external laboratory using a commercial reverse transcription–PCR (RT-PCR) assay.^3^

Human resource records were used to collect baseline characteristics including age, sex, ethnicity, and job role. HCWs working from home, shielding, or on long-term leave were excluded from the analysis. Occupational health records were used to identify HCWs who attended screening and positive COVID-19 cases.

Monthly operational costs included the nursing and administrative support required to collect swabs, couriers, and staff time required to attend screening. Test costs consisted of the test itself plus consumables, including swabs, personal protective equipment (PPE), and stationery. These costs were calculated per month to provide useful scalable information.

This study was approved by The Royal National Orthopaedic Hospital Research and Innovation Committee (RNOH RIC).

### Statistical analysis

We summarized data using descriptive statistics. Categorical data on baseline characteristics were compared using a 2-sided Pearson χ^2^ test. The statistical analysis was performed using SPSS version 26.0 software (IBM, Chicago, IL).

## Results

In total, 2,284 staff were employed by the RNOH during the study period. After exclusions for staff shielding or working from home, 2,045 staff were eligible for screening. Moreover, 2,035 HCWs attended screening at least once, and of these, 967 (47.3%) were fully adherent, 623 (30.5%) were partially adherent, and 455 (22.2%) were nonadherent to serial testing. Of the remaining 10 HCWs, 4 formally refused (3 due to anxiety and 1 due to chronic sinusitis). A summary of demographic characteristics (ie, age, sex, ethnicity, and staff group) is shown in Table 1. In total, 5,204 samples were collected during the study period; 15 (0.29%) were invalid. Of the remaining 5,189 samples, 2 were positive, equating to a positivity rate of 0.04% (2 of 5,204 swabs analyzed) and prevalence of 0.10% (2 of 2,045).

**Table 1.** Comparing Baseline Characteristics and Adherence to Serial to Asymptomatic COVID-19 Screening of HCWs

Baseline Characteristic^a^	Total	Fully Adherent With Screening, No. (%)^b^	Partially Adherent With Screening, No. (%)^c^	Nonadherent With Screening, No. (%)^d^	*P* Value
Total	2,045	967 (47.3)	623 (30.5)	455 (22.2)	
**Sex**					
Male	694	299 (43.1)	221 (31.8)	174 (25.1)	<.001
Female	1,351	668 (49.4)	402 (29.8)	281 (20.8)	
**Race or ethnic group**					
White	1,035	568 (54.9)	293 (28.3)	174 (16.8)	<.001
Asian	567	247 (43.6)	177 (31.2)	143 (25.2)	
Black or Afro-Caribbean	369	115 (31.2)	131 (35.5)	123 (33.3)	
Chinese	14	8 (57.1)	4 (28.6)	2 (14.3)	
Mixed	60	29 (48.3)	18 (30)	13 (21.7)	
**Staff group**					
Administrative and clerical staff	621	310 (49.9)	184 (29.6)	127 (20.5)	<.001
Nursing	421	185 (43.9)	141 (33.5)	95 (22.6)	
Allied health professionals	260	155 (59.6)	67 (25.8)	38 (14.6)	
Clinical support staff	171	85 (49.7)	56 (32.7)	30 (17.5)	
Surgeons and medics	237	121 (51.1)	72 (30.4)	44 (18.6)	
Porters, caterers, and house staff	174	10 (5.7)	70 (40.2)	94 (54)	
Professional, scientific, and technical staff	161	101 (62.7)	33 (20.5)	27 (16.8)	

Note. HCW, healthcare worker.

a For sex, ethnicity and staff group, the Pearson 2-sided *χ* ^2^ test was performed for hypothesis testing, without adjustment for multiple comparisons.

b HCWs with 4 or more tests were categorized as fully adherent.

c HCWs with 2, or 3 tests were categorized as partially adherent.

d HCWs with no tests or attended a single test were categorized as nonadherent,

Theoretical costs for the onsite testing center working at a maximum capacity of 3,200 tests was £70,520 (USD 88,142) per month (Table 2). Actual costs based on an average of 2,602 tests costing £62,443 (USD 78,047) per month, and assuming operational costs remained constant, were estimated to be £749,316 (USD 936,566) per year. The 2 positive cases identified over 8 weeks cost an average of £62,443 (USD 78,047) per case per month.

**Table 2.** Monthly Costs of the Onsite Testing Center^a^

Group	Operational Costs per Month	Test Costs per 3,200
RT-PCR Test	…	42,000
Sample collection	…	720
PPE	…	450
Stationery	…	50
Band 5 nurse (2.0 FTE)	4,400	…
Band 6 nurse (0.5 FTE)	1,200	…
Band 3 administrator (2.0 FTE)	3,000	…
Courier (RNOH to FCI) (200/day)	5,400	…
Staff time to attend the testing center^b^	13,300	…
Subtotal	27,300	43,220
Total cost per 3,200 tests per month	70,520	

Note. RT-PCR, reverse-transcription polymerase chain reaction; FTE, full-time equivalent; RNOH, Royal National Orthopaedic Hospital; FCI, Francis Crick Institute, London.

a Maximum capacity based on the staffing level was 6,400 tests over the 8-week study period.

b Based on 15-min period to attend the onsite center and the median salary midpoint for all staff attending testing.

## Discussion

Multiple snapshot studies demonstrate PCR screening of asymptomatic HCWs can identify positive cases. The increase in number and throughput of PCR tests has led to asymptomatic screening in high-risk clinical settings being advocated.^4^ However, current studies include selected HCW populations over short periods,^5,6^ and it is unclear whether serial screening is acceptable, effective, or worth the resources required in a low-prevalence setting.

Our finding that full adherence to serial screening was 47% is concerning because asymptomatic and presymptomatic cases may go undetected. Low adherence among certain staff groups (eg, nursing and clinical support workers, porters, caterers, and domestic staff) is also worrying because all may come into close contact with patients or their environment. Reasons for low adherence are multifactorial and may be procedural (eg, reliance on staff booking their own appointments or lack of a reminder) or individual (eg, perceptions that screening is not important, vaccine hesitancy, or financial concerns) or may reflect inequity of access.

Among 2,035 HCWs, 2 HCWs (0.10%) tested positive for COVID-19 during the study period. This low rate may be largely explained by the low background rate in the London region during the study period. This finding is consistent with previously published data at our hospital showing that HCW cases closely match the community prevalence.^7^ Both positive HCWs were truly asymptomatic and could potentially have contributed to onward transmission if undetected.

The optimum frequency of testing has not been determined. A modeling study simulated the effect of different PCR testing frequencies on the likelihood of preventing an outbreak for a range of R numbers.^8^ To reduce the R to <1, testing frequency would need to be almost every other day for an R of 2.5, at least twice weekly (every 3–4 days) for an R of 2, or weekly for an R was 1.5. These researchers did not simulate R numbers below 1.5 such as those seen during our study period. These researchers factored in a short turnaround time of 24 hours for a result, which was not achievable in our study with once-daily transport of samples. More frequent testing would enable earlier detection of truly asymptomatic and presymptomatic cases but would increase operational and testing costs.

Screening costs alone would place significant strain on healthcare budgets. Furthermore, the cost of identifying 1 positive asymptomatic case is above the threshold used by National Insitutue of Clinical Excellence to evaluate cost-effectiveness when assessing suitability for funding treatments^9^; however, costs avoided by preventing onward transmission were not estimated.

Our study was limited by its single-center retrospective design. We lacked behavioral data to understand the reasons for poor adherence. Our study was conducted during a time before lateral flow tests were widely available, and these offer significant advantages such as avoiding the need for a testing center, faster turnaround times, and lower costs. However, lateral flow tests have lower sensitivity than PCR, potentially limiting their use for screening. Our study was conducted before vaccination was widely available, which may have reduced the impact of screening. To scale costs, we assumed constant adherence throughout the year.

In conclusion, adherence to PCR screening is poor, with significant differences across ethnicities and staff groups. The resources required to identify a single asymptomatic case per month are substantial and may not be cost-effective during a period of low prevalence. Our findings may provide useful information for policy makers when deciding on resource allocation to facilitate elective surgery.
